# Mesenchymal Stromal Cells: Updates and Therapeutic Outlook in Rheumatic Diseases

**DOI:** 10.3390/jcm2040201

**Published:** 2013-10-23

**Authors:** Yves-Marie Pers, Christian Jorgensen

**Affiliations:** Clinical Immunology and Osteoarticular Diseases Therapeutic Unit, Lapeyronie University Hospital, 371, Avenue du Doyen Gaston Giraud, Montpellier 34295, France; E-Mail: ympers2000@yahoo.fr

**Keywords:** mesenchymal stem cells, rheumatic diseases, osteoarthritis, rheumatoid arthritis, autoimmune diseases, immune tolerance

## Abstract

Multipotent mesenchymal stromal cells or mesenchymal stem cells (MSCs) are adult stem cells exhibiting functional properties that have opened the way for cell-based clinical therapies. MSCs have been reported to exhibit immunosuppressive as well as healing properties, improving angiogenesis and preventing apoptosis or fibrosis through the secretion of paracrine mediators. This review summarizes recent progress on the clinical application of stem cells therapy in some inflammatory and degenerative rheumatic diseases. To date, most of the available data have been obtained in preclinical models and clinical efficacy needs to be evaluated through controlled randomized double-blind trials.

## 1. Introduction

**Figure 1:**
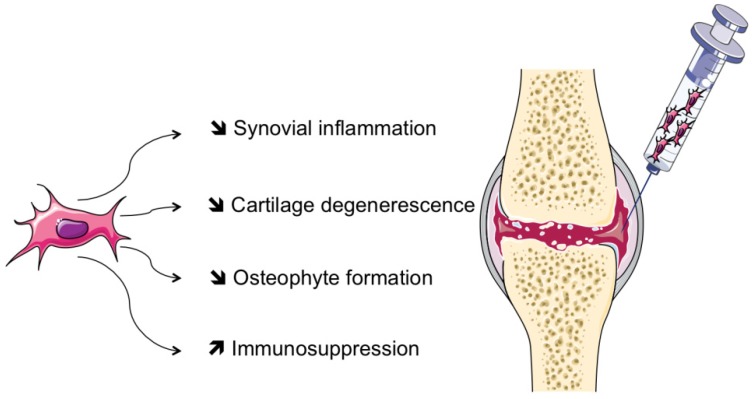
Schematic representation of mesenchymal stem cell (MSC)-based therapy via intra-articular injection in the treatment of rheumatic diseases. Through the release of trophic factors and cell contact, MSCs may act to reduce cartilage degeneration, osteophyte formation and synovial inflammation. MSCs may also have systemic properties that may be of interest in extra-articular manifestations of rheumatic diseases.

Mesenchymal stem cells, or stromal cells (MSCs) are progenitor cells mainly isolated from adult bone marrow (BM), and adipose tissue. MSCs are also present in most adult tissues, including muscles, synovial tissue, placental tissue, and teeth [1]. The last two localizations allow easier collection and therefore an extension of their use. They have several functions: Synthesis of extracellular matrix, immune tolerance, development, inflammation and fibrosis. Each of their properties seems to have promising future therapeutic applications in various diseases (see Figure 1).

MSCs are defined by their functional abilities of differentiation and differ from hematopoietic stem cells by the expression of mesenchymal markers (CD105, CD70, CD90), while lacking expression of CD34, CD45, CD14 monocyte or markers of T or B cells, or the major histocompatibility class II (MHC II) [2,3]. MSCs have a phenotypic heterogeneity with some multipotent properties and are the progenitors of multiple lineages including bone, cartilage, muscle or fat. Understanding the mechanisms of action of MSCs in various diseases can be hampered by their phenotypic heterogeneity since each phenotype may be associated with a different biological response. MSCs are currently being studied for tissue engineering applications, including bone and cartilage repair because of their potential to differentiate into different lineages such as chondrocytes, osteoblasts or adipocytes [3]. Recently, these cells have also been shown to have immunosuppressive and healing capacities, to improve angiogenesis and prevent fibrosis.

## 2. Biological Properties of MSCs

### 2.1. Modulation of the Immune Response

MSCs have immunomodulatory and immunosuppressive properties and are involved in both the innate and the adaptive immunity [1,4,5]. This immunosuppressive effect is mainly due to the secretion of soluble factors by MSCs and by direct contact with immune cells. MSCs acquire their immunosuppressive properties after exposure to an inflammatory environment. Some cytokines such as tumor necrosis factor alpha (TNF-α), interleukin 1 beta (IL-1-β) or interferon gamma (IFN-γ) are able to activate MSCs [6].

Whatever the T cell status (naive or activated), MSCs have a suppressive role in CD4^+^ and CD8^+^ T cells by blocking their cell cycle by inhibiting the expression of cyclin D2. Dendritic cells (DCs) are the key antigen presenting cells. MSCs modulate the immune response because they are able to avoid the transformation of CD34^+^ DC into mature DC (via the co-secretion of IL-4 and granulocyte-macrophage colony-stimulating factor (GM-CSF)) [7]. MSCs also promote a failure of antigen presentation by DCs [7]. MSCs in contact with DC acquire a tolerogenic profile producing a large amount of IL-10 and decreasing the secretion of IL-12 [8]. Finally, MSCs induce the expression of a regulator phenotype (Treg) CD4^+^CD25^+^Foxp3^+^ in different murine models (asthma, diabetes, autoimmune encephalitis, *etc.*). Recently, our team showed that the beneficial effect observed following MSCs injection in an autoimmune mouse model was associated with the suppression of Th17 cells and the increase in CD4^+^CD25^+^Foxp3^+^ T lymphocytes percentage [9].

These suppressive properties of MSCs not only depend on cell contact but also on secretion of regulatory molecules [5]. Adhesion molecules are involved in MSC-mediated immunosuppression. In a MSC and T cell coculture system, activated T cells secrete several inflammatory cytokines, including IFN-γ, TNF-α, and IL-1. The combinations of IFN-γ with TNF-α or IL-1 can upregulate the expression of adhesion molecules: Intercellular adhesion molecule-1 (ICAM-1) and vascular cell adhesion molecule-1 (VCAM-1). Importantly, blocking of the function of ICAM-1 and VCAM-1 significantly reversed the immunosuppressive effect of MSCs *in vitro* and *in vivo* [10]. Programmed death-1 (PD-1) is a molecule expressed on various cell types that plays an important role in the negative regulation of immune responses and the maintenance of peripheral tolerance [11]. Our team demonstrated that MSC suppressive effect on mature Th17 cell function and proliferation is contact-dependent and mediated by PD-1 pathway up-regulation on primed MSCs [12]. Thus, these molecules are important for MSC-mediated immunosuppression through cell-cell interactions.

Some mediators, indoleamine 2,3-dioxygenase (IDO), nitric oxide synthase (iNOS) as well as the secretion of human leukocyte antigen (HLA-G), transforming growth factor (TGFβ), interleukin-6 (IL-6), protein that stimulates the TNFα gene (TSG6) or prostaglandin E2 (PGE2) have been proposed to play a significant role in the suppressive properties of MSCs. Contradictory results have been reported on the implication of IDO in MSC-mediated immunosuppression. While a key role of IDO is suggested by a majority of studies [13,14], human MSCs deficient in the expression of both IFN-γ receptor 1 and IDO activity still exert important immunomodulatory activity [15]. Secretion of nitric oxide (NO), produced by iNOS in MSC has been shown to inhibit T cell proliferation involving a Stat5-dependent pathway [16]. NO is considered as an important cytotoxic effector molecule, the activity of which is dependent on the presence of IFN-γ [17]. Furthermore, MSCs from iNOS −/− mice have a reduced ability to suppress T cell proliferation, both *in vitro* and *in vivo*, in a model of graft-*versus*-host disease and delayed-type hypersensitivity [17].

### 2.2. MSC and Immunogenicity

MSCs seem to be immune privileged cells due to the low expression of MHC and co-stimulation mechanisms of T cell (CD80/CD86, CD40) [4]. Soluble HLA-G also seems to participate in this tolerance [18]. These features help prevent a rapid rejection and immune sensitization. However, transplant rejection of MSCs has been described in the literature in the long term [19]. NK cells may be involved in increasing rejection of allogeneic donor BM cells. MSC had only a partial inhibitory effect on proliferating NK cells. More importantly, activated NK cells could lyse efficiently MSCs [20]. However, MSC may suppress NK activation, and this enhanced by IFN-γ prestimulation. This occurs through multiple mechanisms, including PGE2 secretion [21]. At low concentration of IFNγ or activation by Toll-like receptors (TLR), increased expression of MHC II and stimulation of CD4^+^ proliferation do not affect enough immunogenicity of MSCs [22]. These findings should be taken into consideration when using MSCs in clinical trials, including whether autologous or allogeneic MSCs should be employed.

### 2.3. MSC and Tissue Homeostasis

In tissue repair, MSCs do not seem to have a direct effect, but they stimulate the regenerative properties of resident cells. They have a paracrine effect by reducing the release of pro-inflammatory cytokines and by stimulating the secretion of anti-inflammatory cytokines. MSCs exert their regulatory role by forming a perivascular niche in close contact with endothelial cells and osteoblasts in bone marrow and in close relationship with the immune and hematopoietic stem cells [5,23]. MSCs operate in collaboration with endothelial factors (epidermal growth factor (EGF), vascular endothelial growth factor (VEGF), insulin-like growth factor 1 (IGF-1), stromal cell-derived factor 1 (SDF-1), transforming growth factor (TGF), Angiopoietin 1) to improve their “homing” on damaged sites. The various studies on intravenous injection of MSCs show a redistribution of cells mainly in lungs and a modest uptake by the liver and kidney. However, the biodistribution of MSCs may be modified in some pathological conditions (inflammation, senescence, *etc.*). Kraitchman *et al.* found evidence of trafficking of MSCs to the heart after induction of ischemic lesion suggesting the responsibility of chemotactic factors [24].

## 3. MSC-Based Therapies for Rheumatic Diseases

**Figure 2:**
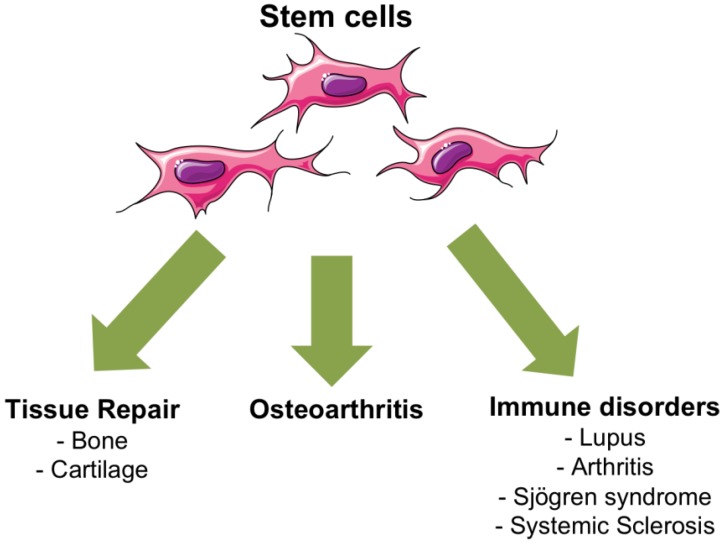
Current therapeutic applications of mesenchymal stem cells (MSCs) in rheumatic diseases.

MSC-based cell therapies represent innovative strategies for the treatment of rheumatic diseases for which currently available treatments are limited and rarely restore the full functions of the tissue. In Figure 2, we summarized some therapeutic applications of MSCs in rheumatic diseases.

### 3.1. MSCs for Osteoarthritis

Osteoarthritis (OA) is a multifactorial disease, quite debilitating, which can affect both weight-bearing joints such as knees and non-bearing joints such as hands. Despite a very high prevalence (12% in the age group >60 years), no effective disease modifying OA drug is available and patients must often have a total joint replacement [25]. OA leads to progressive degeneration of the cartilage, sclerosis of the subchondral bone with formation of osteophytes and subchondral cysts. Severe OA causes chronic pain, stiffness, deformation and effusion, leading to reduced function and quality of life.

The rationale for using local injection of MSCs for inducing regeneration of OA cartilage is based on a first *in vivo* study in a caprine model [26]. Whatever the source of MSCs (BM, adipose tissue or synovium), factors secreted by MSCs increased cartilage matrix production by chondrocytes. Adipose-derived stem cells (ASCs) share many properties with MSCs but may easily be collected through liposuction and be used in human trials. Local injection of BM-MSCs or ASCs in the joint is likely to exert several roles: Inhibition of osteophyte formation, decrease in synovial inflammation, reduction in cartilage degeneration with less fibrosis and apoptosis of chondrocytes or stimulation of chondrocyte proliferation and extracellular matrix synthesis. However, neither the exact mechanism of action when ASCs or BM-MSCs are not in direct contact with chondrocytes, nor the identification of possible mediators, have been investigated.

Although OA is not considered an inflammatory disease, pro-inflammatory mediators, such as cytokines, metalloproteinases (MMP), reactive oxygen species (ROS), are secreted by OA chondrocytes or synoviocytes and participate in joint tissue alterations. Several pro-inflammatory cytokines are significantly down-regulated in chondrocytes when cultured with ASCs suggesting that ASCs may also be protective through the down-regulation of inflammatory mediators [27]. Interestingly, paracrine factors of BM-MSCs share the same anti-inflammatory effects on OA cartilage and synovial explants *in vitro* [28]. Moreover, a significant decrease in TGF-β1 secretion by chondrocytes and induction of Hepatocyte Growth Factor (HGF) secretion by ASCs was observed in OA explants [29].

Recently, new studies have provided interesting data on animal models. In the first work, a single ASC injection in the knee joint of mice showed significant decrease in synovitis score and cartilage damage probably by suppressing synovial macrophage activation [30]. The local benefits have been replicated in a rabbit model of OA induced by meniscectomy and anterior cruciate ligament transection [31]. The cells were found locally up to 6 months after intra-articular injection [32]. Biodistribution and toxicology studies have confirmed safety of ASCs injections [32].

In humans, no controlled studies have been published yet. In order to prevent OA, MSCs have been administered locally in 55 patients undergoing meniscectomy, and absence of local side effects was reported (Chondrogen^®^ Osiris Therapeutics Inc. trial, Columbia, MD, USA). Recently, in Iran, four patients with moderate-to-severe knee OA were selected for a Phase I study [33]. Autologous BM-MSCs were injected in the knee joint. They observed improvement in walking time and reduction in walking pain in three patients. Most importantly, no side effects were reported after 1-year follow-up [33]. Moreover, another Iranian phase I clinical trial recently reported that intra-articular injection of autologous BM-MSCs in six patients with knee OA was safe and improved pain and functional status of the knee. As important, magnetic resonance imaging (MRI) displayed increased cartilage thickness and decreased subchondral oedemas in three out of six patients [34]. All these data support the trophic action of MSCs for protecting cartilage from degradation and stimulating regeneration. A clinical phase I trial has been initiated at the sites of Montpellier (France) and Würzburg (Germany). The principle is based on liposuction, expansion of ASCs and their local autologous injections in knee osteoarthritis. This trial (NCT01585857) has obtained the authorization and agreement of the National Security Agency (ANSM). The study is in progress and preliminary results show excellent tolerance to the local injection. However, due to the low number of patients and the absence of a control group it is too early to draw any conclusion of clinical benefit.

### 3.2. MSC for Rheumatoid Arthritis

Currently, no human clinical trial has been conducted in rheumatoid arthritis (RA). In the collagen-induced arthritis (CIA) murine model, which is representative of RA in humans, contrasted results have been reported. Thus, it has been reported that a single injection of primary murine MSCs prevents the onset of arthritis, which was associated with a decrease in serum pro-inflammatory cytokines and increased Treg [35,36]. The therapeutic benefit of xenogeneic human MSCs, from either adipose tissue or umbilical cord, has also been described [37,38]. However, other studies failed to demonstrate any improvement with MSC treatment. Systemic infusion of the allogeneic C3H10T1/2 cell line increased local inflammation and the clinical signs of arthritis [39]. The immunosuppressive effect of primary murine BM-MSCs seems to depend on genetic backgrounds. In contrast to allogeneic MSCs, syngeneic or partially mismatched MSCs may delay the time of onset of clinical disease and alter disease progression in CIA [40]. Alternatively, the immunomodulatory role of BM-MSCs was reported to be dependent on the window of injection, with therapeutic benefit only when two cell injections on day 18 and 24 were done [36].

### 3.3. MSC for Autoimmune Diseases

#### 3.3.1. Lupus

Systemic Lupus Erythematosus (SLE) is an autoimmune disease with various clinical features that may involve life-threatening, such as renal or neurological manifestations. SLE is characterized by B-cell hyperactivity, autoantibody production as antinuclear, anti-DNA and anti-Sm. Several studies are underway. The first Chinese pilot study was published in 2009 on 4 patients treated with doses of 1 to 10 million cells per kg [41]. Two other cases of refractory SLE treated with autologous MSCs were published [42]. These patients underwent injection of autologous bone marrow MSCs without therapeutic benefit. One patient developed renal failure due to SLE. Tolerance injection was acceptable and the authors observed a slight increase in the circulation of regulatory T cells.

More recently, two phase I/II studies were published in lupus nephritis, one of 15 patients with allogeneic MSCs from BM [43] and the other of 16 patients with MSCs derived from umbilical cord [44]. Most patients improved clinically and serologically, but the follow-up was short and pre-treatment may have affected the results.

Recently, MSCs have been evaluated in 35 SLE patients with refractory cytopenia, of which 20 had leukopenia and 24 had thrombocytopenia [45]. The authors found a significant improvement in haematological parameters for most patients. Clinical remission was accompanied by an increase in Treg and a decrease in Th17. Two patients died during the study.

In all published series, the feasibility and safety are acceptable, although the efficacy remains an unresolved issue. Large prospective randomized studies are needed, particularly in refractory lupus nephritis.

#### 3.3.2. Sjögren’s Syndrome

The immunosuppressive properties of MSCs have also been recently evaluated by Chinese authors in Sjögren’s syndrome (SS). In a beautiful work, Xu J. *et al.* have confirmed in a non-obese diabetic (NOD) mice model the benefit of allogeneic MSCs from bone marrow on the modulation of the immune response by promoting the production of Treg lymphocytes and switch to a Th2 profile [46]. They have highlighted a reduction in the production of Th17 after injection of MSCs. They confirmed these results by injection of allogeneic MSCs, derived from umbilical cord, in a heterogeneous panel of 24 patients affected by SS. Eleven patients had refractory dryness syndrome and 13 had systemic involvement of the disease (thrombocytopenia, anemia, hepatitis, tubulopathy, interstitial pneumonia, enteritis and neurological complications). Although patient characteristics, associated treatment during follow-up and the lack of control arm can be criticized, it is the first study suggesting clinical efficacy (visual analog scale (VAS) patient activity score EULAR Sjögren’ syndrome disease activity index (ESSDAI), salivary flow) of MSCs in the Sjögren’s syndrome. In addition, it is very interesting to note biological effectiveness with a sharp and rapid decrease in the production of anti-SSA antibodies.

#### 3.3.3. Systemic Sclerosis

Systemic sclerosis (SSc) is a rare disease where therapeutic benefit can be expected because of the pro-angiogenic and anti-fibrotic effects mediated by MSCs. MSCs isolated from the bone marrow of patients with SSc have differentiation properties similar to MSCs from healthy donor and maintain their immunosuppressive properties on T cells [47]. In these patients, the ability of MSCs to differentiate into endothelial progenitor cells seems reduced as migration functions and pro-angiogenic potential of these progenitors [48]. These data support on the use of autologous or allogeneic MSCs in this disease.

Currently, we have few observations of the use of MSCs in patients with SSc. One patient with severe SSc, refractory to conventional treatments, was treated by intravenous injection of allogeneic MSCs [49]. Three months after injection, a significant decrease in the number of ulcers was observed. At 6 months, blood flow to the hands and fingers and transcutaneous partial pressure of oxygen were significantly improved. Rodnan skin score was reduced from 25 to 11. On the other hand, the benefit of the administration of autologous MSC was recently described in one patient by obtaining a regression of the distal necrosis surfaces after the first injection of MSCs [50].

Recently, Keyszer *et al.* reported observations of five patients with refractory SSc who received the injection of MSCs [51]. The treatment appears to be relatively well tolerated. Although their results were quite heterogeneous, it made them consider phase I–II clinical trials in this disease. In France, a National Hospital Clinical Research Program coordinated by D. Farge (Saint-Louis, MO, USA, Paris, France) was held in 2011 and is expected to begin shortly.

## 4. Conclusions

MSC-based cell therapies represent innovative strategies for the treatment of rheumatic diseases for which currently available treatments are limited and rarely restore the full functions of the tissue. Several studies are ongoing. We have listed in Table 1 all the studies currently under recruitment and referenced in ClinicalTrials.gov registry. We look forward to the results because we need to evaluate clinical efficacy of stem cells through controlled randomized double-blind trials.

**Table 1:** Summary of studies currently under recruitment in ClinicalTrials.gov using MSC as a therapeutic option in rheumatic diseases.

Diseases	Type of stem cells	Localization	Autologous or allogeneic	Phase study	ClinicalTrials.gov identifier	Date of study first received	Sponsor country
OA	ASC	IA Knee	Autologous	I	NCT01585857	March 2012	France
OA	BM-MSC	IA Knee	Allogeneic	I–II	NCT01586312	April 2012	Spain
OA	BM-MSC	IA Knee	Autologous	II	NCT01459640	October 2011	Malaysia
RA	MPC	IV	Allogeneic	I–II	NCT01851070	April 2013	USA
RA	UC-MSC	IV	Allogeneic	I–II	NCT01547091	February 2012	China
SLE	UC-MSC	IV	Allogeneic	I–II	NCT01741857	November 2012	China
SLE nephritis	UC-MSC	IV	Allogeneic	II	NCT01539902	February 2012	China
AS	UC-MSC	IV	Allogeneic	I	NCT01420432	August 2011	China

OA: osteoarthritis; RA: rheumatoid arthritis; SLE: systemic lupus erythematosus; AS: ankylosing spondylitis; ASC: adipose stem cell; BM: bone marrow; MSC: mesenchymal stem cell; MPC: mesenchymal precursor cell; UC: umbilical cord; IA: intra-articular; IV: intra-venous.

New therapeutic applications of MSCs aim at interfering with immune responses of patients in various inflammatory autoimmune disorders or inhibiting progress of the clinical symptoms in degenerative diseases. Besides current research on mechanisms regulating the therapeutic efficacy of MSCs, more knowledge on migration, biodistribution, survival and safety of MSCs need to be obtained for generalized therapeutic use in rheumatic diseases.
